# Loss of ASXL1 in the bone marrow niche dysregulates hematopoietic stem and progenitor cell fates

**DOI:** 10.1038/s41421-017-0004-z

**Published:** 2018-01-23

**Authors:** Peng Zhang, Zizhen Chen, Rong Li, Ying Guo, Hui Shi, Jie Bai, Hui Yang, Mengyao Sheng, Zhaomin Li, Zhuo Li, Jianping Li, Shi Chen, Weiping Yuan, Tao Cheng, Mingjiang Xu, Yuan Zhou, Feng-Chun Yang

**Affiliations:** 1 Department of Biochemistry and Molecular Biology, University of Miami Miller School of Medicine, Miami, FL 33136 USA; 2 Sylvester Comprehensive Cancer Center, University of Miami Miller School of Medicine, Miami, FL 33136 USA; 3 State Key Laboratory of Experimental Hematology, Institute of Hematology and Blood Diseases Hospital and Center for Stem Cell Medicine, Chinese Academy of Medical Sciences and Peking Union Medical College, Tianjin, 300020 China

## Abstract

Somatic or de novo mutations of *Additional sex combs-like 1* (*ASXL1*) frequently occur in patients with myeloid malignancies or Bohring-Opitz syndrome, respectively. We have reported that global loss of *Asxl1* leads to the development of myeloid malignancies and impairs bone marrow stromal cell (BMSC) fates in mice. However, the impact of *Asxl1* deletion in the BM niche on hematopoiesis remains unclear. Here, we showed that BMSCs derived from chronic myelomonocytic leukemia patients had reduced expression of *ASXL1*, which impaired the maintaining cord blood CD34^+^ cell colony-forming capacity with a myeloid differentiation bias. Furthermore, *Asxl1* deletion in the mouse BMSCs altered hematopoietic stem and progenitor cell (HSC/HPC) pool and a preferential myeloid lineage increment. Immunoprecipitation and ChIP-seq analyses demonstrated a novel interaction of ASXL1 with the core subunits of RNA polymerase II (RNAPII) complex. Convergent analyses of RNA-seq and ChIP-seq data revealed that loss of *Asxl1* deregulated RNAPII transcriptional function and altered the expression of genes critical for HSC/HPC maintenance, such as *Vcam1*. Altogether, our study provides a mechanistic insight into the function of ASXL1 in the niche to maintain normal hematopoiesis; and *ASXL1* alteration in, at least, a subset of the niche cells induces myeloid differentiation bias, thus, contributes the progression of myeloid malignancies.

## Introduction

The Drosophila Asx protein belongs to the enhancer of Trithorax and Polycomb group and functions in both transcriptional activation and repression^1,2^. Trithorax and Polycomb proteins have significant impacts on various biological processes by modifying chromatin structures to control the active/repressive transcriptional states, respectively^3^. There are three Asx homologs in mammals, additional sex combs-like 1 (ASXL1), ASXL2, and ASXL3^4^. Three ASXL members share conserved domains, including N-terminal ASXN, ASXH domains, and a C-terminal plant homeodomain^4^. As a chromatin regulator, ASXL1 plays an important role in epigenetic regulation by activating or repressing the transcription of genes involved in either differentiation or proliferation through its effect on histone methylation marks^5,6^. ASXL1 has been shown as an essential cofactor for the histone H2A deubiquitinase BAP1^6^, as well as a critical mediator of the function of polycomb repressive complex 2 (PRC2)^5^. Recently, we reported that ASXL1-cohesin interaction functions as a novel way to maintain normal sister chromatid separation and to regulate gene expression in hematopoietic cells^7^. These studies demonstrate multifaceted functions of ASXL1 in gene regulation by assembling epigenetic regulators and transcription factors to specific gene loci.

Genomic sequencing studies have uncovered an array of distinct genomic driver mutations in various cancers, including myeloid malignancies. *ASXL1* mutations are often found in a wide range of myeloid malignancies^8–11^, and its alterations are associated with poor prognosis^12^. Hoischen et al.^13^ reported that de novo* ASXL1* mutations occur in patients with Bohring-Opitz syndrome (BOS) and some of these patients develop Wilms tumors^14^. We and others have established mouse models and verified that loss of *Asxl1* leads to myelodysplastic syndrome (MDS)-like disease^15,16^ and BOS-like phenotypes^17^. We also showed that ASXL1 regulates the self-renewal and differentiation of bone marrow stromal cells (BMSCs)^17^ and hematopoietic stem/progenitor cells (HSC/HPCs)^15,16^.

HSC/HPCs reside in the bone marrow (BM), known as BM “niche”. The normal function of the BM niche is critical for the maintenance of cellular function of HSC/HPCs^18–23^. BMSCs are the major component of the BM niche that maintain and regulate the HSC/HPC pool throughout life^24,25^. Two independent studies using different mouse models revealed that systemic deletion of *Asxl1* (*Asxl1*^*−/−*^) leads to severer hematologic phenotypes^16^ than the conditional loss of *Asxl1* in hematopoietic cells alone^15^. This led us to hypothesize that *Asxl1* loss in the niche of *Asxl1*^*−/−*^ mice contributes to the hematopoietic phenotypes in vivo. Biased myeloid differentiation prerequisites leukemia formation^26^. Furthermore, preferential expansion of the granulocyte-macrophage progenitor (GMP) population is associated with a high risk of leukemic transformation in MDS patients^27,28^. Given the fact that global deletion of *Asxl1* results in biased myeloid differentiation, we questioned that *Asxl1*-deficient niche may alter the cell fates of HSC/HPCs, contributing to the pathogenesis of myeloid malignancies.

In the current study, we found that the expression of *ASXL1* significantly decreased in the BMSCs of chronic myelomonocytic leukemia patients (CMML-BMSCs) compared with healthy donors (HD-BMSCs). In addition, CMML-BMSCs displayed a reduced hematopoietic supportive activity and induced a skewed HSC/HPC differentiation toward granulocytic/monocytic lineage. Furthermore, utilizing *OsxCre;Asxl1*^*fl/fl*^ mouse model, we showed that deletion of *Asxl1* in the BM niche impaired HSC/HPC pool and skewed cell differentiation with a bias to granulocytic/monocytic lineage. Interestingly, immunoprecipitation assays showed that ASXL1 interacted with the core subunit of RNA polymerase II (RNAPII) complex, POLR2A, in BMSCs. Chromatin immunoprecipitation followed by DNA sequencing (ChIP-seq) analyses identified a co-occupancy of ASXL1 and RNAPII at the gene promoter regions. Loss of *Asxl1* reduced RNAPII enrichment genome-wide accompanied by altered expression of genes critical for BMSC self-renewal, differentiation, and biological functions. Our study provides a further mechanistic insight into ASXL1 functions in the BM niche, and how *ASXL1* alteration-associated defective niche works in concert with an intrinsic effect of *ASXL1* alteration-mediated HSC/HPC defects to promote the pathogenesis of myeloid malignancies.

## Results

### Reduced CFU-F frequency and decreased proliferative capacity in CMML-BMSCs

**Fig. 1:**
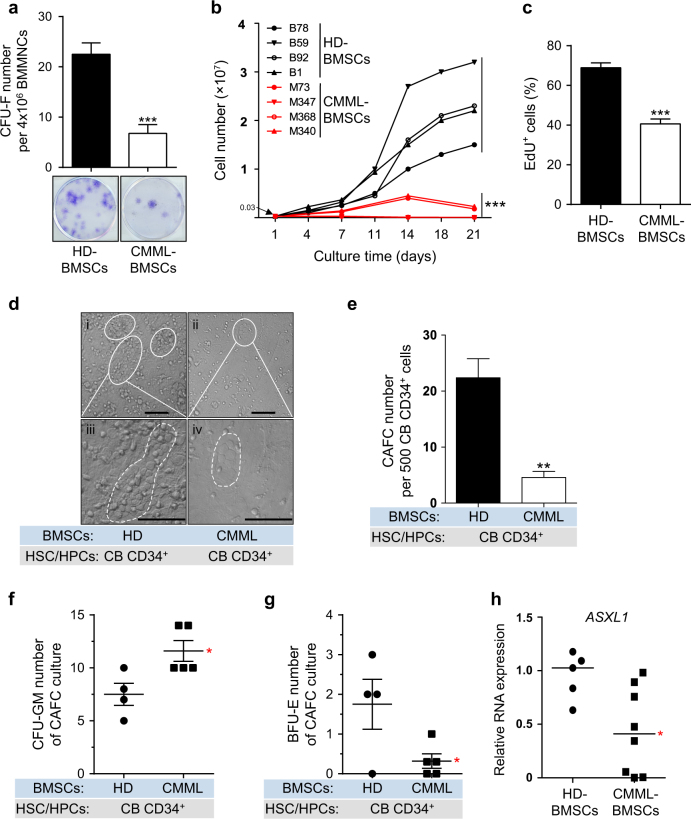
CMML-BMSCs exhibit decreased proliferative capacity and reduced hematopoietic supportive activity. **a** The frequency of CFU-F from CMML patients shows a dramatically decreased BMSC pool compared with HD controls after 10 days of culture (*n* = 5 individual samples for each group). Representative photographs of CFU-F are shown. **b** Growth curves for 21 days shows reduced proliferation ability of CMML-BMSCs (red lines, *n* = 4) compared with HD-BMSCs (black lines, *n* = 4). Each dot represents one passage. **c** Bar graph shows the percentage of EdU^+^ cells (*n* = 4 individual samples for HD, 3 for CMML). **d** Long-term co-culture of CB CD34^+^ cells with CMML-BMSCs or HD-BMSCs indicates impaired hematopoietic supportive ability of CMML-BMSCs. Representative micrographs of CAFCs after 5 weeks of co-culture with HD-BMSCs (i, iii) or CMML-BMSCs (ii, iv) are shown. Scale bar, 50 µm. **e** The numbers of CAFC are reduced in CB CD34^+^ cells co-cultured with CMML-BMSCs. Quantitative evaluation of CAFC number per 500 CB CD34^+^ cells is shown (*n* = 4 individual samples for HD, 5 for CMML). **f**,** g** After co-culture of CB CD34^+^ cells with HD-BMSCs or CMML-BMSCs, the CFU-C assays were performed. The colony numbers of CFU-GM (**f**) and BFU-E (**g**) are shown (*n* = 4 individual samples for HD, 5 for CMML). **h** qPCR analysis shows that *ASXL1* transcripts were significantly decreased in CMML-BMSCs compared with HD-BMSCs (*n* = 5 individual samples for HD, 8 for CMML). Bars represent the median. Data represent mean ± s.e.m., * *P* < 0.05. **P < 0.01, ***P < 0.001

BMSCs from thirteen CMML patients and ten healthy donors were isolated and cultured in vitro. The clinical characteristics of CMML patients were listed in Supplementary Table S1. CMML-BMSCs exhibited similar morphology and expression pattern of cell surface markers as in HD-BMSCs (Supplementary Fig. S1a, b). Colony-forming unit-fibroblast (CFU-F) assay revealed a significant reduction in the frequency of CFU-F in the BM of CMML patients compared with that in the HD-BM (Fig. 1a), indicating a reduced BMSC pool in the BM of CMML patients.

To determine the growth characteristics of CMML-BMSCs and HD-BMSCs, cell numbers were assessed dynamically in the cultures of BMSCs. CMML-BMSCs exhibited limited proliferative capacity during passaging in vitro compared with HD-BMSCs (Fig. 1b). EdU staining further confirmed the impaired proliferation in the CMML-BMSCs. After incubation with EdU for 36 h, ~70% of HD-BMSCs were EdU-positive (EdU^+^), while only 40% CMML-BMSCs were EdU^+^ (Fig. 1c; Supplementary Fig. S1c). Cell cycle analysis revealed a marked reduction in S-phase of CMML-BMSCs compared with HD-BMSCs (Supplementary Fig. S1d, e). In addition, senescence-associated β-galactosidase staining showed a threefold increase in the percentage of senescent cells in CMML-BMSCs compared with HD-BMSCs (Supplementary Fig. S1f). Collectively, these data indicate that CMML-BMSCs have a decreased proliferative capacity, an increased senescent activity, and reduced DNA replication.

BMSCs are capable of differentiating into osteoblasts^29^. CMML-BMSCs exhibited a less alkaline phosphatase-positive expression (Supplementary Fig. S1g), as well as decreased mRNA expression of multiple genes (*ALPL*, *BGLAP*, and *RUNX2*) critical for osteoblast differentiation compared with HD-BMSCs (Supplementary Fig. S1h). These data suggest an impaired osteoblast differentiation in CMML-BMSCs.

### Reduced hematopoietic supportive activity and increased granulomonocytic lineage differentiation

BMSCs are widely recognized to be important in HSC maintenance within the BM microenvironment^24^. Given that CMML-BMSCs exhibited impaired proliferative capacity and cell fate determination, we next examined the hematopoietic supportive activity of CMML-BMSCs using cobblestone-area-forming cell (CAFC) assays in vitro. A significant reduction of CAFC number and smaller CAFC sizes were observed in the co-culture of cord blood (CB) CD34^+^ cells with CMML-BMSCs compared with HD-BMSCs (Fig. 1d, e). These data indicate that CMML-BMSCs have an impaired hematopoietic supportive activity.

To examine the effect of CMML-BMSCs on the differentiation of normal HSC/HPCs in vitro, we performed long-term culture-initiating cell (LTC-IC) assays. An increased number of CFU-granulocytes/macrophages (CFU-GM) was observed in the co-culture of CB CD34^+^ cells with CMML-BMSCs compared with HD-BMSCs (Fig. 1f). In contrast, the number of burst forming unit-erythrocytes (BFU-E) was significantly decreased in the co-culture of CB CD34^+^ cells with CMML-BMSCs compared with HD-BMSCs (Fig. 1g). These results demonstrate that the co-culture of CB CD34^+^ cells with CMML-BMSCs preferentially promotes myeloid differentiation.

### Decreased *ASXL1* expression in CMML-BMSCs

Mutations of *ASXL1* frequently occurred in the hematopoietic cells of CMML patients^12^. To examine whether the CMML-BMSCs carry *ASXL1* mutation, targeted PCR followed by Sanger sequencing was performed on genomic DNA extracted from the CMML-BMSCs. No *ASXL1* mutation was identified in the BMSCs of these 13 CMML patients who have *ASXL1* mutations in the hematopoietic cells (data not shown). However, the mRNA expression levels of *ASXL1* were significantly decreased in the cohort of CMML-BMSCs (Fig. 1h).

To determine whether the decreased *ASXL1* expression is a direct cause of the functional alteration of the patient BMSCs, we next introduced WT ASXL1 into BMSCs derived from myeloproliferative neoplasms (MPN) patients. Quantitative PCR (qPCR) confirmed that *ASXL1* mRNA was overexpressed in MPN-BMSCs compared with the empty vector (EV) controls (Supplementary Fig. S1i). After 7 days of co-culture of MPN-BMSCs with CB CD34^+^ cells, *ASXL1* overexpression in MPN-BMSCs restored their effect on CB CD34^+^ cell differentiation as evidenced by the reduced percentage of myeloid cells (CD45^+^/CD33^+^) and increased percentage of erythroid cells (CD71^+^/CD235a^+^) compared with EV-transduced MPN-BMSCs (Supplementary Fig. S1j, k).

### Altered cell fates of HSC/HPCs in *OsxCre;Asxl1*^*fl/fl*^ mice

We and others reported that loss of *Asxl1* in mice led to MDS-like disease^15,16^. Interestingly, global deletion of *Asxl1* leads to severer hematologic phenotypes^16^ than the conditional loss of *Asxl1* in hematopoietic cells alone^15^. Our previous study showed that loss of *Asxl1* in mice led to a marked decrease in the number of BMSCs compared with WT mice; and *Asxl1*^*−/−*^ BMSCs exhibited impaired self-renewal and skewed differentiation^17^. We argued that deletion of *Asxl1* in the BM niche could cooperate with *Asxl1-*null HSC/HPCs to accelerate the pathogenesis of myeloid malignancies in vivo. To test this hypothesis, we performed hematopoietic phenotypic analyses in *OsxCre;Asxl1*^*fl/fl*^ mice, in which *Asxl1* is deleted in the BMSCs and preosteoblasts^30,31^. Of note, BMSCs and preosteoblasts have been considered as important components of BM niche^32^. The successful *Asxl1* deletion in BMSCs was shown by PCR (Supplementary Fig. S2a), and the expression of *Asxl1* was not affected in lineage negative/cKit^+^ (LK) cells (Supplementary Fig. S2a, b). The von Kossa/McNeal staining of the *OsxCre;Asxl1*^*fl/fl*^ femur revealed a decreased trabecular bone surface and an increased adipocyte number (Supplementary Fig. S2c–e).

**Fig. 2:**
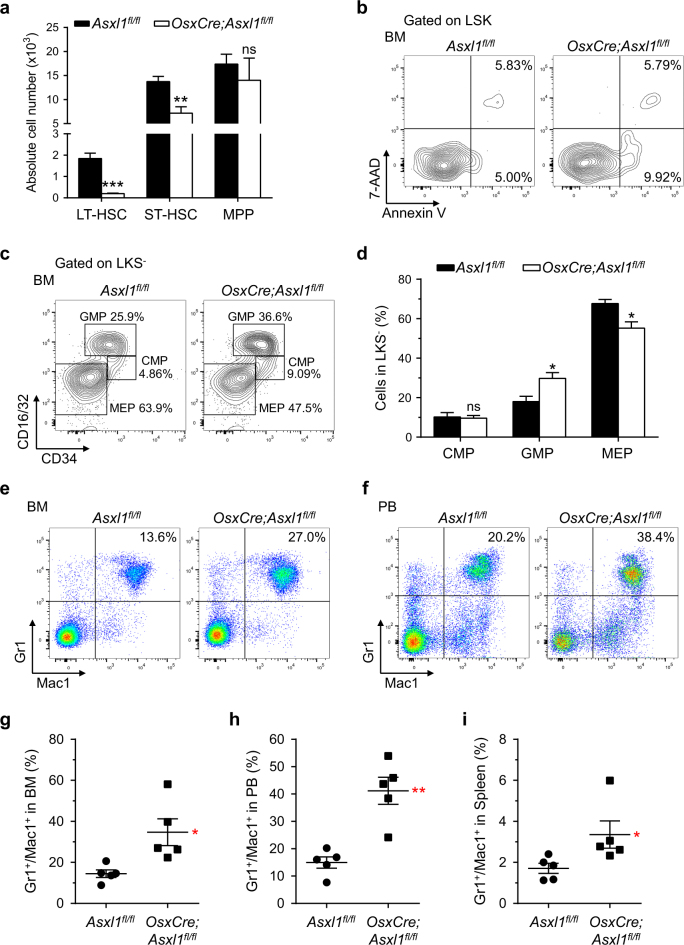
Altered HSC/HPC and myeloid populations in *OsxCre;Asxl1*^*fl/fl*^ mice. **a** Absolute numbers of LT-HSCs, ST-HSCs, and MPP cells are shown (*n* = 4 mice per genotype). **b** Apoptosis analysis (Annexin V/7-AAD staining) on freshly isolated LSK cells from BM of representative *Asxl1*^*fl/fl*^ and *OsxCre;Asxl1*^*fl/fl*^ mice. **c** Flow cytometric analysis of CMP, GMP, and MEP populations in the Lin^−^cKit^+^Scal1^−^ (LKS^−^) cells from BM of representative *Asxl1*^*fl/fl*^ and *OsxCre;Asxl1*^*fl/fl*^ mice. **d** Quantification of the percent GMP and MEP populations in LKS^−^ cells of *OsxCre;Asxl1*^*fl/fl*^ mice are shown (*n* = 4 mice per genotype). **e**,** f** Flow cytometric analysis of Gr1^+^/Mac1^+^ cell populations in BM (**e**) and PB (**f**) of representative *Asxl1*^*fl/fl*^ and *OsxCre;Asxl1*^*fl/fl*^ mice. **g–i** Quantification of Gr1^+^/Mac1^+^ cell populations in BM (**g**), PB (**h**), and spleen (**i**) revealed increased granulocytic/monocytic cells in *OsxCre;Asxl1*^*fl/fl*^ mice (*n* = 5 mice per genotype). Data represent mean ± s.e.m., ns, not significant, *P < 0.05, **P < 0.01, ***P < 0.001

To assess whether deletion of *Asxl1* in the BM niche affects the HSC/HPC pool, we performed phenotypic analyses on the BM cells of *Asxl1*^*fl/fl*^ (control) and *OsxCre;Asxl1*^*fl/fl*^ mice by flow cytometry. The number of long-term HSCs (LT-HSCs) and short-term HSCs (ST-HSCs) per femur was dramatically reduced in *OsxCre;Asxl1*^*fl/fl*^ mice compared with *Asxl1*^*fl/fl*^ control mice (Fig. 2a). *OsxCre;Asxl1*^*fl/fl*^ mice had a significantly lower proportion of LT-HSCs in BM Lin^−^Sca1^+^cKit^+^ (LSK) cells compared with control mice (Supplementary Fig. S2f, g). The ST-HSCs and multipotential progenitor (MPP) cell populations in the BM LSK cells of *OsxCre;Asxl1*^*fl/fl*^ mice were similar to that of *Asxl1*^*fl/fl*^ mice (Supplementary Fig. S2f, g).

The changes in the HSC/HPC frequencies in vivo can be associated with altered apoptosis. We then examined whether *Asxl1* loss in the BM niche affects the survival of HSC/HPCs by flow cytometric analyses following Annexin V and 7-amino-actinomycin D (7-AAD) staining. *OsxCre;Asxl1*^*fl/fl*^ LSK cells had a significantly higher proportion of Annexin V^+^/7-AAD^−^ cells than *Asxl1*^*fl/fl*^ cells (Fig. 2b; Supplementary Fig. S2h). These results indicate that loss of *Asxl1* in the BM niche increases HSC/HPC apoptosis, which may contribute to the lower numbers of LT-HSCs and ST-HSCs in vivo.

Further analyses showed that deletion of *Asxl1* in the BM niche significantly increased GMP population and decreased megakaryocyte-erythrocyte progenitor (MEP) population, whereas common myeloid progenitor (CMP) population was similar in the BM Lin^−^cKit^+^Sca1^−^ (LKS^−^) cells of *OsxCre;Asxl1*^*fl/fl*^ mice compared with control mice (Fig. 2c, d). However, the number of GMP, MEP, and CMP cell populations was similar between the two genotypes of mice (Supplementary Fig. S2i). To further confirm the effect of *Asxl1* loss in BM niche on HSC/HPCs, we performed the CFU-C assays using the BM of *Asxl1*^*fl/fl*^ and *OsxCre;Asxl1*^*fl/fl*^ mice. The frequency of CFU-GM was significantly higher in the BM of *OsxCre;Asxl1*^*fl/fl*^ mice than in the BM of *Asxl1*^*fl/fl*^ control mice (Supplementary Fig. S2j), suggesting a preferential myeloid progenitor increment.

To further characterize the *Asxl1* loss in BM niche-mediated dysregulation of hematopoiesis, flow cytometric analyses were performed on peripheral blood (PB), BM, and spleen cells of *Asxl1*^*fl/fl*^ and *OsxCre;Asxl1*^*fl/fl*^ mice. An increased proportion of granulocytic/monocytic cells (Gr1^+^/Mac1^+^) was observed in the PB, BM, and spleens of *OsxCre;Asxl1*^*fl/fl*^ mice compared with control mice (Fig. 2e–i).

Given that *Asxl1*^*−/−*^ BMSCs exhibit impaired self-renewal and skewed differentiation^17^, we sought to explore the role of *Asxl1* in BMSCs in the maintenance of HSC/HPC activity. We began by performing CAFC assay to evaluate the hematopoietic supportive activity of *Asxl1*^*−/−*^ BMSCs. When LK cells were co-cultured for 4 weeks on BMSC feeder layers, the numbers of CAFC and CFU-C were significantly reduced in the co-culture of *Asxl1*^*−/−*^ BMSCs plus WT LK cells compared with WT co-cultures (Supplementary Fig. S3a, b). This result suggests that *Asxl1*^*−/−*^ BMSCs had an impaired hematopoietic supportive activity. To further assess the effect of *Asxl1*^*−/−*^ BMSCs in hematopoietic cell differentiation, the percentage of Gr1^+^/Mac1^+^ cells in the co-cultures was analyzed by flow cytometry. After 2 weeks of co-culture, *Asxl1*^*−/−*^ BMSCs induced a biased Gr1^+^/Mac1^+^ differentiation of WT LK cells compared with WT BMSCs (Supplementary Fig. S3c), verifying that *Asxl1*^*−/−*^ BMSCs mediates a biased myeloid differentiation of HSC/HPCs.

### Loss of *Asxl1* in the BM niche alters HSC/HPC fate

**Fig. 3:**
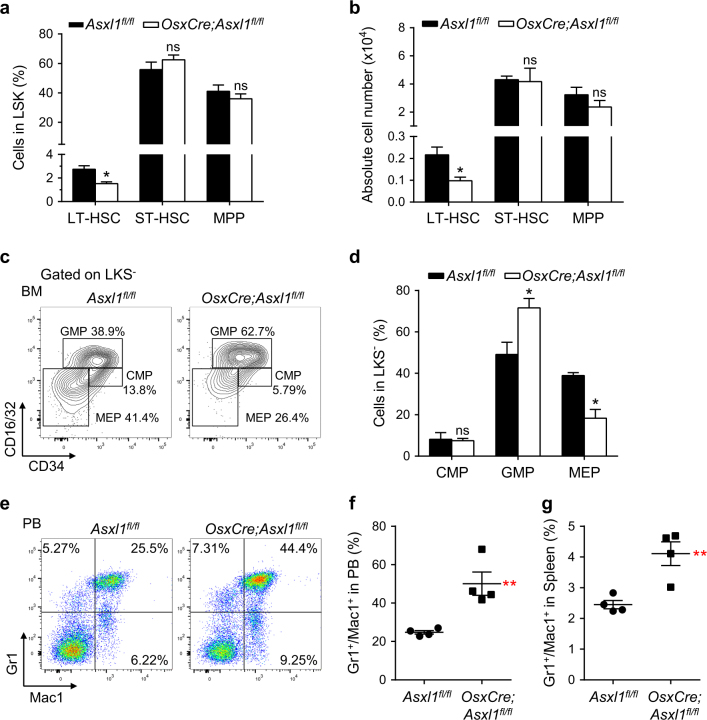
Loss of *Asxl1* in the BM niche alters HSC/HPC pool. **a** Frequencies of LT-HSC, ST-HSC, and MPP cell populations in BM reconstituted CD45.1^+^/LSK cells of *OsxCre;Asxl1*^*fl/fl*^ recipients are shown (*n* = 3 mice per genotype). **b** Absolute numbers of LT-HSCs, ST-HSCs, and MPP cells from *Asxl1*^*fl/fl*^ and *OsxCre;Asxl1*^*fl/fl*^ recipients are shown (*n* = 3 mice per genotype). **c** Flow cytometric analyses of CMP, GMP, and MEP populations in the reconstituted CD45.1^+^/LKS^−^ cells from BM of representative *Asxl1*^*fl/fl*^ and *OsxCre;Asxl1*^*fl/fl*^ recipients. **d** Quantification of the percent CMP, GMP, and MEP populations in BM reconstituted CD45.1^+^/LKS^−^ cells of *OsxCre;Asxl1*^*fl/fl*^ recipients are shown (*n* = 3 mice per genotype). **e** Flow cytometric analysis of Gr1^+^/Mac1^+^ cell populations in PB of representative *Asxl1*^*fl/fl*^ and *OsxCre;Asxl1*^*fl/fl*^ recipients. **f**,** g** Quantification of Gr1^+^/Mac1^+^ cell populations in PB (**f**) and spleen (**g**) shows increased myeloid cells in *OsxCre;Asxl1*^*fl/fl*^ recipients (*n* = 4 mice per genotype). Data represent mean ± s.e.m., ns, not significant, *P < 0.05, **P < 0.01

To assess the contribution of the microenvironment to the hematopoietic phenotypes in *OsxCre;Asxl1*^*fl/fl*^ mice, we next transplanted WT BM (CD45.1^+^) cells into lethally irradiated *Asxl1*^*fl/fl*^ and *OsxCre;Asxl1*^*fl/fl*^ recipient mice, respectively. Five months after the transplantation, we performed flow cytometric analyses to determine the frequencies of HSC/HPCs and the myeloid cell population of reconstituted cells (CD45.1^+^) (Supplementary Fig. S3d). Interestingly, the frequency and number of LT-HSCs were significantly decreased in *OsxCre;Asxl1*^*fl/fl*^ recipients compared with the control recipients (Fig. 3a, b; Supplementary Fig. S3e). Furthermore, significantly increased GMP and decreased MEP populations were observed in BM LKS^−^ cells of *OsxCre;Asxl1*^*fl/fl*^ recipients compared with that of control recipients (Fig. 3c, d). However, the number of GMP population in *OsxCre;Asxl1*^*fl/fl*^ recipients was comparable to that in the control recipients, whereas the number of MEP was significantly decreased (Supplementary Fig. S3f). *OsxCre;Asxl1*^*fl/fl*^ recipients had a significantly high proportion of Gr1^+^/Mac1^+^ cells in the PB and spleens (Fig. 3e–g; Supplementary Fig. S3g). The results reinforce the impact of *OsxCre;Asxl1*^*fl/fl*^ microenvironment in altering the HSC/HPC pool and promoting myeloid differentiation bias in vivo.

To further examine whether the impaired HSC/HPCs in *OsxCre;Asxl1*^*fl/fl*^ mice are transplantable, we transplanted *Asxl1*^*fl/fl*^ and *OsxCre;Asxl1*^*fl/fl*^ BM cells into lethally irradiated WT-recipient mice, respectively. Histological analyses of the femur at 6 months post transplantation revealed an accumulation of myeloid cells in the recipients transplanted with *OsxCre;Asxl1*^*fl/fl*^ BM cells compared with the recipients transplanted with *Asxl1*^*fl/fl*^ BM cells (Supplementary Fig. S3h). In addition, the area with adipocytes was larger in *OsxCre;Asxl1*^*fl/fl*^ BM transplanted recipients compared with *Asxl1*^*fl/fl*^ BM transplanted recipients (Supplementary Fig. S3h). The increase in adipocyte expansion may be caused by the following reasons: (1) a less proliferative potential of donor HSC/HPCs (*OsxCre;Asxl1*^*fl/fl*^ BM) may reduce the marrow cellularity, resulting in an expansion of adipocytes in the BM; (2) the impaired HSC/HPCs of *OsxCre;Asxl1*^*fl/fl*^ donor mice produce altered growth factors, which dysregulate cell fates of BMSCs, leading to adipocyte accumulation. Collectively, these results suggest that the *Asxl1*-deficient niche may mediate permanent marks in hematopoietic cells.

### Loss of *Asxl1* in BMSCs dysregulates genes required for HSC/HPC maintenance

**Fig. 4:**
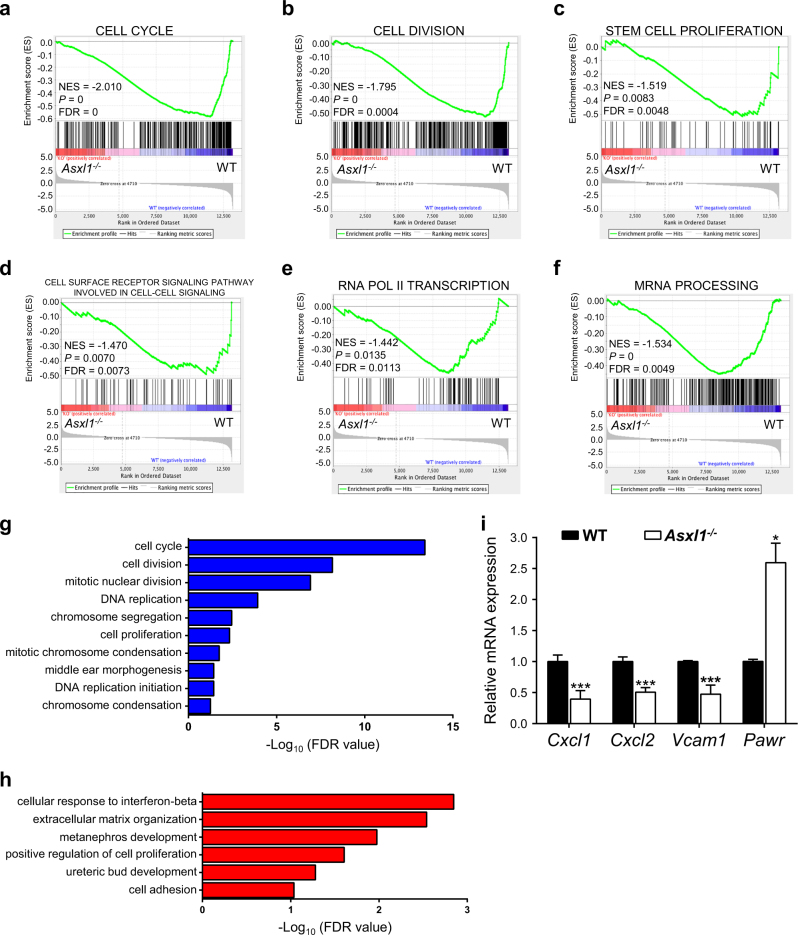
Loss of *Asxl1* in BMSCs alters the expression of genes associated with the HSC/HPC maintenance. **a–f** GSEA plots showing downregulated genes in *Asxl1*^*−/−*^ BMSCs significantly enriched in the cell cycle (**a**), cell division (**b**), stem cell proliferation (**c**), cell surface receptor signaling pathway involved in cell–cell signaling (**d**), and mRNA transcription (**e**,** f**) signature. The normalized enrichment score (NES), *P*-value and false discovery rate (FDR) are shown. **g**,** h** GO analysis of the down- (**g**, blue) and up- (**h**, red) regulated genes in *Asxl1*^*−/−*^ BMSCs compared with WT cells (FDR < 0.1). **i** qPCR shows dysregulated genes expression in *Asxl1*^*−/−*^ BMSCs compared with WT BMSCs (*n* = 3~5 mice for WT, *n* = 4~6 mice for *Asxl1*^*−/−*^). Data represent mean ± s.e.m., *P < 0.05, ***P < 0.005

We have previously reported that *Asxl1* loss dysregulated transcriptional programs to induce lineage commitment of BMSCs^17^. To examine if the dysregulated genes in *Asxl1*^*−/−*^ BMSCs are associated with the HSC/HPC maintenance, we re-analyzed the RNA-seq data (GSE75787)^17^. Gene set enrichment analysis (GSEA) showed that the downregulated genes in *Asxl1*^*−/−*^ BMSCs were enriched for cell cycle, cell division, stem cell proliferation, cell surface receptor signaling pathway involved in cell–cell signaling, and mRNA transcription signatures (Fig. 4a–f). Gene ontology (GO) analyses revealed that the downregulated genes were associated with cell cycle, cell division, and cell proliferation (Fig. 4g). In contrast, the upregulated genes were associated with cellular response to interferon-beta, extracellular matrix organization, and cell adhesion (Fig. 4h). Interestingly, the genes critical for the function of the BM niche on hematopoiesis, such as *Vcam1, Cxcl1*^33^, and *Cxcl2*^34^, were downregulated in *Asxl1*^*−/−*^ BMSCs (Fig. 4i; Supplementary Fig. S4a). These data suggest that *Asxl1* is required for normal BM niche activity to maintain normal hematopoiesis. Further studies are warranted to investigate the underlying mechanisms by which *ASXL1* alteration dysregulates the BM niche, contributing to the impaired HSC/HPC function.

### ASXL1 functionally associates with RNAPII complex

**Fig. 5:**
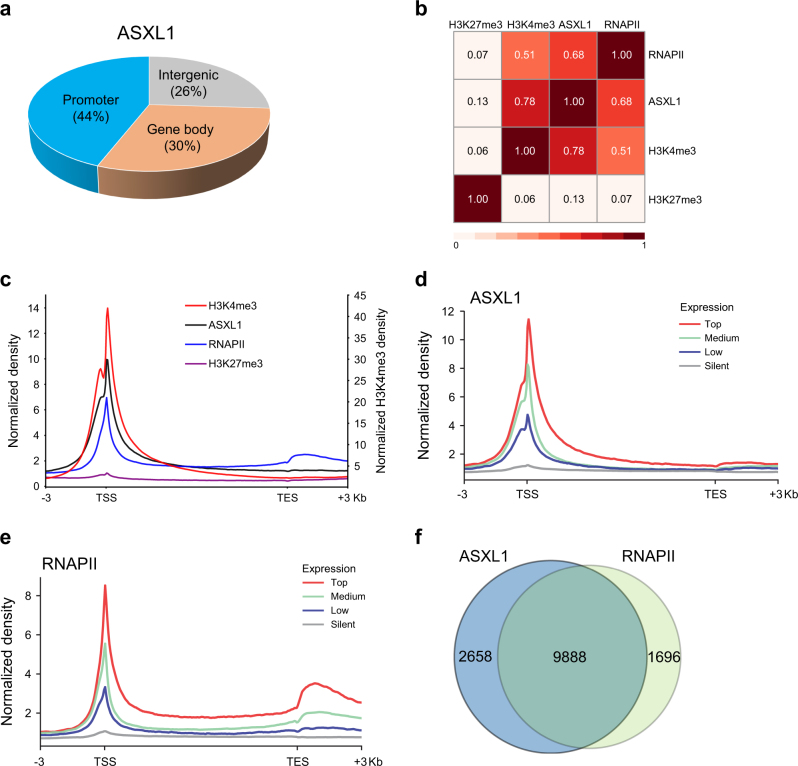
ASXL1 co-localizes with RNAPII in BMSCs. **a** Genomic distribution of ASXL1 peaks in WT BMSCs. **b** Heatmap showing a pairwise correlation between ASXL1, H3K4me3, H3K27me3, and RNAPII in WT BMSCs. Pearson correlation coefficients are given for each comparison. **c** Average genome-wide occupancies of H3K4me3 (red, right *y*-axis), ASXL1 (black), RNAPII (blue), and H3K27me3 (purple) on ASXL1-bound genes along the transcription unit in WT BMSCs. **d**,** e** ASXL1 (**d**) and RNAPII (**e**) occupancies across genes classified by expression. The gene body length is aligned by percentage from the TSS to transcription end site (TES). 3 kb upstream of TSS and 3 kb downstream of TES are also included. **f** Venn-diagram shows the overlap among target genes of ASXL1 with RNAPII in WT BMSCs

We and others have reported that ASXL1 is mainly located at transcription start sites (TSSs) on the genome of hematopoietic cells^7,15^. To determine the ASXL1-associated regions on the genome of BMSCs, ChIP-seq assay was performed using anti-ASXL1 antibody. We identified 24,347 ASXL1-binding sites in WT BMSCs. Genome-wide mapping of the ASXL1-binding regions showed forty-four percent of ASXL1-binding sites were localized at the promoter regions (± 3 kilobase (kb) of TSSs, Fig. 5a), suggesting an association of ASXL1 in gene regulation. ASXL1 was reported to bind to PRC2 complex and loss of *Asxl1* reduced the global levels of tri-methylation of histone H3 lysine 27 (H3K27me3) and H3K4me3 in myeloid cells^5,15,16^. To determine the putative impact of ASXL1-mediated H3K27me3 and H3K4me3 on BMSC cell fates, we then performed H3K27me3, H3K4me3, and RNAPII ChIP-seq using WT and *Asxl1*^*−/−*^ BMSCs. Surprisingly, we did not find a strong association (*r* = 0.13) between ASXL1 binding and H3K27me3 enrichment on the genome of BMSCs (Fig. 5b, c; Supplementary Fig. S4b, c). Furthermore, a minimal correlation (*r* = 0.07) was observed between H3K27me3 enrichment and RNAPII occupancy (Fig. 5b). In contrast, a significant positive association (*r* = 0.78) was observed between ASXL1 binding and H3K4me3 enrichment (Fig. 5b, c; Supplementary Fig. S4c). However, loss of *Asxl1* in BMSCs did not affect the H3K4me3 enrichment on the genome of BMSCs (Supplementary Fig. S4d, e). These results suggest ASXL1 regulates gene expression in an H3K27me3/H3K4me3-independent manner in BMSCs.

It is recognized that increased gene transcription correlates with increase dynamic supercoiling and demands more recruitment of specific genes^35,36^. It is possible that more ASXL1 can be found in the promoter regions of active genes. At genes with high (75–100%), medium (25–75%), low (bottom 25% of genes with significant output), or silent (genes with nonsignificant output) expression as measured by RNA-seq, the density of ASXL1 peaks was indeed associated with gene expression levels (Fig. 5d). The distribution of ASXL1 at promoter regions was comparable to that of RNAPII in BMSCs (Fig. 5e). To examine whether ASXL1 and RNAPII co-localize at the same genomic regions, integrative analyses were performed to assess the genome-wide distribution in BMSCs. The results showed a significant positive correlation between ASXL1 binding and RNAPII occupancy (*r* = 0.68, Fig. 5b). ASXL1 promoter binding was highly overlapped with RNAPII loading (Fig. 5c). Importantly, analyses of the coverage between ASXL1 and RNAPII bound genes showed a high degree (85.36%) of overlay (Fig. 5f; Supplementary Fig S4f, g). These data suggest a role for ASXL1 involving in gene regulation.

### ASXL1 interacts with RNAPII in BMSCs

**Fig. 6:**
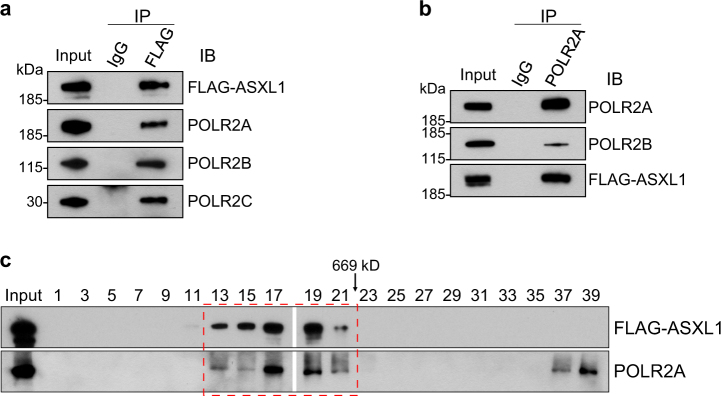
ASXL1 interacts with RNAPII complex in BMSCs. **a** Nuclear extracts of WT BMSCs transduced with FLAG-tagged ASXL1 immunoprecipitated (IP) with anti-FLAG or non-immune IgG and probed for FLAG, POLR2A, POLR2B, and POLR2C. **b** Reciprocal IP and western blotting confirmed interaction of ASXL1 with POLR2A, and POLR2B. Nuclear extracts were subjected to IP using POLR2A antibody. **c** Gel filtration analysis of nuclear extracts from FLAG-ASXL1 overexpressing cells. ASXL1 and RNAPII complex were co-eluted from a Superose 6 column as analyzed by western blotting. The numbers over the lanes represent the eluted fraction numbers

To test whether ASXL1 and RNAPII interact and co-localize in the nucleus of BMSCs, we generated a stable BMSC line that constitutively expresses a FLAG-tagged ASXL1 (FLAG-ASXL1) to overcome the limitation of commercially available ASXL1 antibodies (Supplementary Fig. S4h) and then performed immunoprecipitation (IP) assays. We used anti-FLAG antibody to pull down ASXL1 in WT BMSCs, followed by western blots using antibodies against POLR2A, POLR2B, and POLR2C, the subunits of RNA polymerase II complex. Indeed, ASXL1 associated with endogenous POLR2A, POLR2B, and POLR2C (Fig. 6a). In addition, the reciprocal co-IP using POLR2A antibody further verified the interaction between ASXL1 and RNAPII family members (Fig. 6b). When the fractionated BMSC nuclear extracts by gel filtration chromatography were subjected to western blot analysis, we observed a co-elution of ASXL1 and RNAPII in the high-molecular-weight fractions (Fig. 6c). These data indicate that ASXL1 interacts with RNAPII complex in the nucleus of BMSCs.

### Loss of *Asxl1* alters the target gene expression by deregulating RNAPII transcriptional activity

**Fig. 7:**
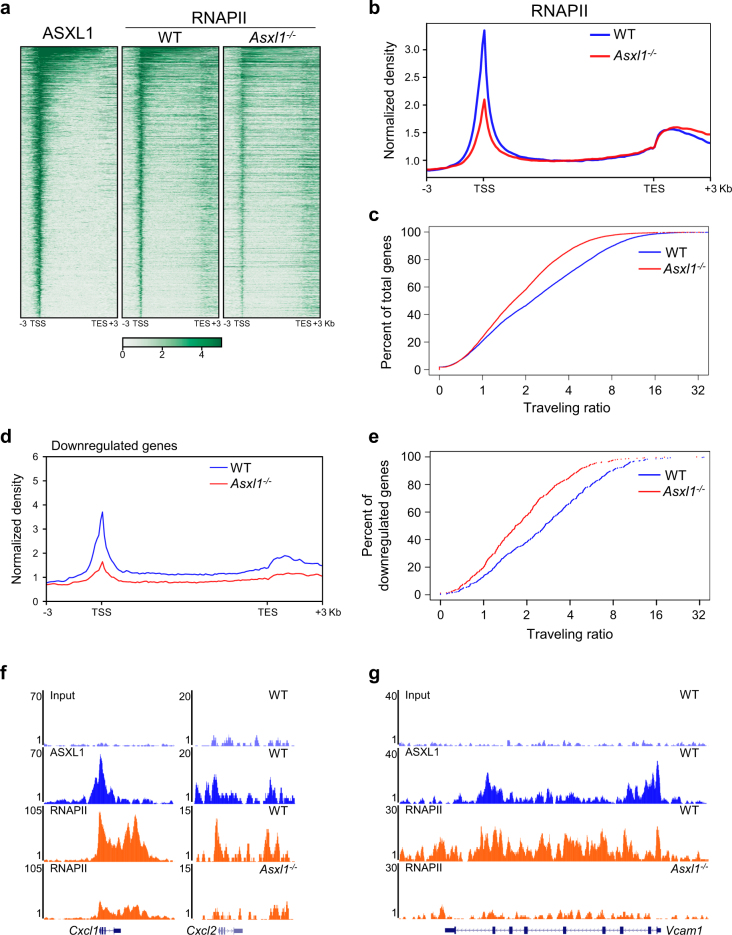
Loss of *Asxl1* dysregulates transcriptional program through RNAPII transcriptional activity. **a** Heatmaps of ASXL1 and RNAPII on ASXL1-bound genes in WT and *Asxl1*^*−/−*^ BMSCs. The genes ranked from highest to lowest ASXL1 level. **b** *Asxl1* deletion reduces genome-wide RNAPII occupancy. **c** RNAPII traveling ratio (TR) shows that many genes become less paused in *Asxl1*^*−/−*^ BMSCs compared with WT controls. Lower TR values indicate a lower degree of pausing. **d** Average genome-wide occupancies of RNAPII in WT (blue) and *Asxl1*^*−/−*^ (red) BMSCs on downregulated genes along the transcription unit. **e** RNAPII TR calculations of downregulated genes in WT and *Asxl1*^*−/−*^ BMSCs. **f**,** g** Representative genome browser tracks showing ASXL1 and RNAPII occupancies on regions of *Cxcl1*, *Cxcl2*, and *Vcam1*

To examine whether loss of *Asxl1* affects RNAPII transcriptional activity, we generated heatmaps using ASXL1 and RNAPII ChIP-seq data (Fig. 7a; Supplementary Fig. S5a). The density of RNAPII peaks was significantly decreased, especially on ASXL1-binding genes in *Asxl1*^*−/−*^ BMSCs compared with WT BMSCs (Fig. 7a, b; Supplementary Fig. S5a, b). The traveling ratio (TR) or the pausing index represents the relative ratio of RNAPII density in the promoter region and the gene body^37–39^. To further characterize RNAPII occupancy on the genome of WT and *Asxl1*^*−/−*^ BMSCs, we calculated the relative ratio of RNAPII density in the promoter region and the gene body (Supplementary Fig. S5c). A substantial shift in TR was observed in *Asxl1*^*−/−*^ BMSCs compared with WT BMSCs, suggesting a decrease in RNAPII density at the transcribed region (Fig. 7c). These results indicate an impact of ASXL1 in regulating RNAPII transcription in BMSCs.

We have previously shown that loss of *Asxl1* in BMSCs altered BMSC self-renewal capacity and lineage commitment with a dysregulated transcriptional program^17^. To correlate the RNAPII status with the dysregulated genes in *Asxl1*^*−/−*^ BMSCs, we next performed convergent analyses on RNA-seq and ChIP-seq data. Decreased RNAPII occupancy at transcribing regions was strongly associated with changes in the mRNA expression for the set of downregulated genes in *Asxl1*^−/−^ BMSCs (Fig. 7d, e). Importantly, ~ 51% of the downregulated genes were ASXL1-binding genes (Supplementary Fig. S5d), which had significant changes of RNAPII occupancy compared with the genes without ASXL1 binding (Supplementary Fig. S5e, f). GO analysis revealed that these ASXL1-binding genes were associated with cell cycle, mRNA transcription, cell division, and stem cell maintenance (Supplementary Fig. S5g). Interestingly, the downregulated genes identified by RNA-seq and qPCR, such as *Vcam1*, *Cxcl1*, and *Cxcl2*, were ASXL1 target genes and had decreased RNAPII signals in *Asxl1*^−/−^ BMSCs (Fig. 7f, g).

Similarly, the upregulated genes were also associated with the RNAPII transcriptional activity in *Asxl1*^−/−^ BMSCs (Supplementary Fig. S6a). About 44% of the upregulated genes were ASXL1-binding genes, which had significant changes of RNAPII enrichment compared with the genes without ASXL1 binding (Supplementary Fig. S6b). The upregulated *Pawr*, an ASXL1 target, had increased RNAPII density in *Asxl1*^−/−^ BMSCs (Supplementary Fig. S6c). Of note, *Runx2* and *Cebpa*, two key genes controlling osteoblast and adipocyte differentiation were ASXL1 target genes, and their expressions were strongly associated with RNAPII occupancy (Supplementary Fig. S6d, e). Collectively, these data suggest that ASXL1 regulates gene expression by regulating RNAPII transcriptional activity.

## Discussion

Somatic mutations of *ASXL1* are frequently found in myeloid malignancies^40^, and loss of *Asxl1* in mice leads to MDS-like diseases^15,16^. Interestingly, systemic deletion of *Asxl1* leads to severer hematologic phenotypes than the conditional loss of *Asxl1* in hematopoietic cells alone. This led us to hypothesize that loss of *Asxl1* in BM niche cooperated with *Asxl1-*deleted HSC/HPCs to contribute the pathogenesis of myeloid malignancies in vivo. Here, we show that ASXL1 in niche cells is required to maintain HSC/HPC functions, and *Asxl1* loss in BMSCs alters the HSC/HPC pool and induces a biased differentiation toward granulocytic/monocytic lineages.

Over the past decade, mutations in epigenetic regulators in the hematopoietic cells have been discovered to be broadly involved in the pathogenesis of myeloid malignancies^40,41^. However, initiation and progression of myeloid malignancies can also be accelerated by the disruption of the architecture and cellular components in the BM niche^32,42–46^. As a vital component of the hematopoietic microenvironment, BMSCs are essential for HSC maintenance and regulation of blood production. Geyh et al.^47^ reported that impaired BMSCs could contribute to hematopoietic insufficiency. In addition, several groups have reported that the BMSCs generated from patients with different myeloid malignancies displayed cellular and functional alterations^48,49^. For example, BMSCs derived from MDS patients appeared to be impaired in immunomodulatory and supporting hematopoiesis function^50^. Recently, Arranz et al.^51^ have demonstrated that depletion of Nestin^+^ BMSCs or their production of CXCL12 may accelerate the progression of MPNs both in vivo and in vitro.

Inoue et al.^52^ have reported that the truncated ASXL1 protein can be detected in MDS cells. They also found that C-terminal deletion mutants of ASXL1 induced an MDS-like disease in the transplanted mice^53^. In the current study, although CMML-BMSCs displayed impaired cellular functions, we did not identify *ASXL1* mutation (a cohort of 13 patients) using targeted PCR followed by Sanger sequencing. Further studies to screen the *ASXL1* mutations with a larger cohort of patients are warranted. *ASXL1*, *TET2*, and *NF1* are known to be important in hematopoiesis^54,55^. We next performed qPCR to compare mRNA levels of *ASXL1*, *TET2*, and *NF1* between CMML-BMSC and HD-BMSC. Interestingly, significant downregulation of *ASXL1* was found in CMML-BMSCs compared with HD-BMSCs, while the expression of *TET2* was identical between these two types of BMSCs. It has been reported that SOX2 directly binds to ASXL1 promoter regions and positively regulates ASXL1 expression^56^. Inoue et al.^57^demonstrated that USP7, a deubiquitinating enzyme, plays an important role in maintaining stabilization of ASXL1. Knockdown of USP7 reduced ASXL1 expression in HL60 cells. However, the mechanism of dysregulated *ASXL1* expression in CMML-BMSCs requires further investigation.

Deletion of *Asxl1* in the BM niche (*OsxCre;Asxl1*^*fl/fl*^) significantly increased the GMP population with skewed differentiation favoring the granulocytic/monocytic lineages. Ye and colleagues^26^ recently showed that skewed myeloid differentiation is a prerequisite for leukemic stem cell formation and leukemia development. Consistently, skewed expansion of GMP population is associated with higher risks of leukemic transformation in MDS patients^27,28^. The hematopoietic phenotypes observed in *OsxCre;Asxl1*^*fl/fl*^ mice indicate that ASXL1 is required for the maintenance of normal function of the BM niche, and *Asxl1* loss in the niche may contribute to the pathogenesis of myeloid malignancies.

The phenotype and cell fate of a given cell rely on the precise control of gene expression by complex transcriptional and epigenetic networks, which is essential for proper differentiation, cellular function, development, and homeostasis^58,59^. Although extensive studies have focused on the identification of intrinsic targets of transcription factors regulating BMSC cell fate and functions, the epigenetic events that control BMSC identity and/or functions remain largely unknown. Here, our data demonstrate that ASXL1 works in concert with RNAPII complex to maintain normal gene expression, and loss of *Asxl1* impaired the genome-wide occupancy of RNAPII. We found that deletion of *Asxl1* in BMSCs dramatically deregulated RNAPII transcriptional activity and resulted in the up- and downregulation of genes critical for BMSC cell fates and biological functions. The interaction between ASXL1 and the core subunits of RNAPII complex supports the notion of ASXL1 involving in gene regulation. We and others have previously reported that loss of *Asxl1* reduced global H3K27me3 levels in myeloid progenitor cells^5,15,16^. To our surprise, we did not observe a significant positive correlation between ASXL1 and H3K27me3 enrichment on the genome of BMSCs, suggesting a cell-lineage dependent association between ASXL1 and interacting partners in the nucleus.

Collectively, our results demonstrate that ASXL1 in the BM niche is required for normal hematopoiesis and *Asxl1* deletion in BMSCs alters HSC/HPC pool and leads to a myeloid differentiation bias. These data provide a mechanistic basis for ASXL1 functions in the BM niche to maintain normal hematopoiesis; and *ASXL1* alteration in, at least, a subset of the niche cells induces myeloid differentiation bias, thus, contributes the progression of myeloid malignancies. This work identifies a novel ASXL1-binding partner, RNAPII protein complex, through which ASXL1 loss leads to dysregulated gene expression. Future efforts are warranted to investigate if *ASXL1* mutation/dysregulation in the BM niche of patients contributes to the pathogenesis of myeloid malignancies, which will shed light on identifying novel therapeutic strategies for patients with *ASXL1* alterations.

## Materials and methods

### Mouse models and reagents

The generation of *Asxl1*^*fl/fl*^ mice have been previously described^15^. *Osx-Cre* transgenic mice were purchased from Jackson Laboratory. All of the murine models were bred on a C57BL/6 genetic background. All studies were conducted in accordance with the regulatory guidelines by the Institutional Animal Care and Use Committee at the University of Miami Miller School of Medicine. Chemicals were obtained from Sigma unless otherwise indicated.

### Patients

Thirteen patients with CMML (ten males and three females) and ten healthy donors (seven males and three females) were included in this study. The study was approved by the Ethics Committee of Institute of Hematology and Blood Diseases Hospital, Chinese Academy of Medical Sciences according to guidelines of the 1975 Helsinki Declaration, and informed consent was received according to the institute’s guidelines on the use of human subjects. All patients were reevaluated and met the 2008 WHO diagnostic criteria.

### Isolation and expansion of BMSCs

Whole BM cells from human CMML patients and healthy donors were cultured at 4 × 10^6^ cells/well in 6-well plate at 37 °C, 5% CO_2_, 5% O_2_ in a fully humidified atmosphere in expansion medium Dulbecco’s modified Eagle medium (DMEM)/F12 (Gibco), supplemented with 10% fetal bovine serum (FBS, Hyclone), 1x insulin transferrin selenium-A (Life Technologies), 10 ng/mL human epidermal growth factor (EGF, Peprotech), and 10 ng/mL human platelet derived growth factor-BB (PDGF-BB, Peprotech). After 10 days’ culture, CFU-fibroblasts (CFU-F) were counted to measure the frequency of BMSCs in the BM following Wright-Giemsa staining as previously reported^17^ and photographs were taken by a Fujifilm digital camera (FinePix 2400Zoom, Fujifilm, Tokyo, Japan). For functional analysis, cells were trypsinized and replated upon reaching 80% confluence and BMSCs at passage three to five were used for the following experiments. Generation of full-length *ASXL1* complementary DNA overexpressing BMSCs were performed as previously described^17^.

### Phenotypic analysis of BMSC surface markers

The phenotypic analyses of BMSCs were performed by evaluating the expression of surface markers on a BD LSR II flow cytometer (BD Biosciences). In brief, BMSCs were incubated with antibodies against CD45 (FITC), CD34 (APC), CD73 (PE), CD105 (APC), CD44 (APC-H7), and CD29 (PE-Cy5) (BD PharMingen) at 4 °C for 30 min. The cells were then washed three times with PBS containing 0.1% bovine serum albumin (BSA) and analyzed by flow cytometry.

### Differentiation assay

To induce osteogenic differentiation, BMSCs were plated at 5 × 10^4^/well in osteogenic differentiation medium, which is DMEM/F12 supplemented with 10% FBS, 10^−8^ M dexamethasone, 10 mM β-glycerophosphoric acid, and 100 μM ascorbic acid in 6-well plate for 1 week. Cells were stained with Alkaline Phosphatase Kit according to the manufacturer’s instruction.

### Cell proliferation assays

To study the proliferative ability of the BMSCs, cell growth was evaluated by manually counting at each passage. Briefly, 3 × 10^5^ BMSCs were seeded in the 60 mm^2^ dish and passaged once the culture reached 80–90% confluence. Cell numbers were recorded and cells were replated at the same density each time. Cell proliferation was also examined by EdU assay. In brief, 1.5 × 10^5^ BMSCs were plated in 20 mm glass bottom dish (NEST Biotechnology, China) and cultured overnight. After incubation with EdU for another 36 h, the cells were fixed in 4% paraformaldehyde for 15 min at room temperature, washed twice with 3% BSA in PBS, incubated with 0.5% Triton X-100 in PBS for 20 min and stained with the Click-iT EdU Imaging Kit (Life Technologies) according to the manufacturer’s instruction. Nuclear staining was performed with 4′, 6-diamidino-2-phenylindole (DAPI). Photomicrographs were taken by Laser confocal fluorescence microscopy (Leica, Germany). The percentage of proliferating cells was calculated based on counting EdU-positive cells/total cells in five individual visual fields.

### Senescence assay

Histochemical staining for senescence-associated β-galactosidase activity was used to measure the senescence of BMSCs. BMSCs were plated in 6-well plate at 5 × 10^4^ cells/well and cultured for 12 h at 37 °C, 5% CO_2_, and 5% O_2_. Cells were then stained with the Senescence Staining Kit (Beyotime Institute of Biotechnology, China) according to the manufacturer’s instructions. Senescent cells displayed a blue staining in the cytoplasm. The percentage of senescent cells was calculated based on counting β-galactosidase-positive cells/total cells in five individual visual fields.

### Cell cycle analysis

Cell cycle analysis was performed using an APC BrdU Flow Kit (BD PharMingen). In brief, 2 × 10^5^ cells were seeded in a T25 flask and cultured for 12 h, BrdU was added 2 h before culture termination, and then nuclear staining was performed with 7-amino-actinomycin D (7-AAD) according to the manufacturer's instruction. Data were acquired on BD LSR II and analyzed by FlowJo.

### Long-term co-culture of HSC/HPCs with BMSCs

The function of BMSCs to support cord blood (CB) CD34^+^ cells was measured by cobblestone-area-forming cell (CAFC) assay and LTC-IC assay. In brief, mononuclear cells of CB were separated by Ficoll-Hypaque density gradient centrifugation. CD34^+^ cells were purified by using magnetic microbeads following the manufacturer’s instructions (Miltenyi, Germany). BMSCs (7000 cells/well) were irradiated with 20 Gy after reaching 90% confluence, and 500 CB CD34^+^ cells were inoculated on top of BMSCs in Iscove modified Dulbecco medium (IMDM, Gibco) supplemented with 10% horse serum (Gibco), 10% FBS, 10^−6^ M hydrocortisone, incubating at 33 °C and 5% CO_2_. After 5 weeks of weekly half-media replacement, the areas containing phase-dim hematopoietic clones (at least five cells) beneath the BMSCs layer were counted as CAFCs. LTC-IC assay was performed by plating all the cells from one co-culture well in H4435 methylcellulose (STEMCELL Technologies, Canada) in one well of a 24-well plate for 14 days.

### Phenotypic analyses of the hematologic system in mice

PB was collected by retro-orbital bleeding and total white blood cells were obtained after lysis of PB with red cell lysis buffer. Single-cell suspensions from BM, spleen, and PB were stained with panels of fluorochrome-conjugated antibodies (Supplementary Table S2). Flow cytometric analysis of HSC/HPCs was performed as previously described^60^. Dead cells were excluded by DAPI staining. The analyses were performed using a BD FACS Canto II or LSR Fortessa flow cytometer. For cell apoptosis analysis, freshly isolated BM cells were stained with the PE-Annexin V/7-AAD Apoptotic Kit (BD Biosciences) according to the protocol and analyzed with LSK subpopulation. All data were analyzed by FlowJo_-_V10 software. For CFU assays, BM cells were plated in triplicate in methylcellulose medium (Methocult M3134, STEMCELL Technologies) supplemented with mouse stem cell factor (100 ng/mL), human interleukin 6 (50 ng/mL), mouse interleukin 3 (5 ng/mL), erythropoietin (4 U/mL), thrombopoietin (100 ng/mL), granulocyte-macrophage-colony-stimulating factor (10 ng/mL, Peprotech) and scored on day 7 of the cultures^61^.

### Gene expression analysis

Total RNA was extracted with TRIzol reagent (Invitrogen), and 1 µg RNA was used for each reverse transcription PCR amplifications with the QuantiTect Reverse Transcription Kit (Qiagen) according to the manufacturer's instructions. qPCR was performed in triplicate using an ABI 7500 with the Fast SYBR green master mix (Applied Biosystems). Mouse *β-actin* and human *GAPDH* were used as the reference genes. Primers used are listed in Supplementary Table S3 and gene expression was calculated by the 2^−ΔΔCt^ method.

### Immunoprecipitation (IP) and western blot

IP was performed using nuclear fraction buffer and antibodies (monoclonal mouse anti-FLAG, Sigma, F3165, clone M2, 1:200; and monoclonal mouse anti-POLR2A, ThermoFisher Scientific, MA1-46093, clone 4H8, 1:200). After washing with IP buffer (20 mM Tris-HCl, pH 7.5, 150 mM NaCl, 1% Triton X-100, 5 mM EDTA, 2 mM sodium orthovanadate, 1 mM phenylmethylsulfonyl fluoride, 2 mM NaF, and protease inhibitor cocktail) four times, the associated proteins were collected for western blot analysis. Antibodies against the following proteins were used: anti-FLAG (1:1000); anti-POLR2A (1:1000); polyclonal rabbit anti-POLR2B (ThermoFisher Scientific, PA5-21446, 1:500); and polyclonal rabbit anti-POLR2C (Abcam, ab138436, 1:2000).

### Gel filtration chromatography

The nuclear extracts were prepared and concentrated to ~ 0.2 mL, and then loaded onto a superose 6 GL 10/300GL column (GE Healthcare) equilibrated at 4 °C with buffer A (20 mM Tris-HCl, 75 mM NaCl, pH 7.8) using an AKTApurifer machine (GE Healthcare). The sample was eluted with buffer A. The column fractions (1 mL each) were subjected to concentrate with Amicon Ultra-0.5 mL Centrifugal Filters (Millipore) and analyzed by western blot.

### RNA-seq analysis

Cuffdiff^62^ was used to detect the differentially expressed genes with a cutoff of *P* < 0.05 and fold change >2. The identified differentially expressed genes were used for pathway enrichment analysis and functional annotation of the Database for Annotation, Visualization, and Integrated Discovery (DAVID) bioinformatics resources^63^. GSEA^64^ was performed with gene signatures in GSEA/MSigDB v6.0, including KEGG pathway signatures and GO signatures. Enriched gene sets or pathways were selected using a cutoff of *P* < 0.05 and FDR < 0.25.

### Chromatin immunoprecipitation assays

BMSCs were fixed with 1% formaldehyde for 15 min and quenched with 0.125 M glycine. Chromatin was isolated and sonicated to an average length of 300 to 500 bp. An aliquot of chromatin was precleared with protein A agarose beads (Invitrogen). Genomic DNA regions of interest were isolated using ASXL1 (Santa Cruz #sc-85283), RNAPII (Abcam #ab5095), H3K4me3 (Active Motif #39159), and H3K27me3 (Millipore #07-449) antibodies. Complexes were eluted from the beads and subjected to RNase and proteinase K treatment. Crosslinks were reversed by incubation overnight at 65 °C, and ChIP DNA was purified by phenol–chloroform extraction and ethanol precipitation. Illumina sequencing libraries were prepared and sequenced on NextSeq 500. The sequence reads were aligned to the mouse genome (mm9) using the BWA algorithm (default settings). ASXL1, RNAPII, H3K4me3, and H3K27me3 peaks were called using the MACS2 program^65^ with default settings. The resulting histograms (genomic “signal maps”) were stored in bigWig files. The UCSC genome browser was used for data visualization. Heatmap and profile plot were generated by deepTools^66^. The accession number for the ChIP-seq data is NCBI GEO: GSE99103.

### Statistical analysis

Differences between experimental groups were determined by Student’s *t*-test or analysis of variance followed by Newman-Keuls multiple comparison tests as appropriate. *P*-values of <0.05 were considered significant.

## Electronic supplementary material

Supplementary information
