# Supplementary material for: Loss of ASXL1 in the bone marrow niche dysregulates hematopoietic stem and progenitor cell fates
Source: Cell Discov. 2018 Jan 23;4:4. doi: 10.1038/s41421-017-0004-z (PMC5802628; doi:10.1038/s41421-017-0004-z)
Supplement: Supplementary file 1 — Supplementary information [file 41421_2017_4_MOESM1_ESM.pdf]

Supplementary information

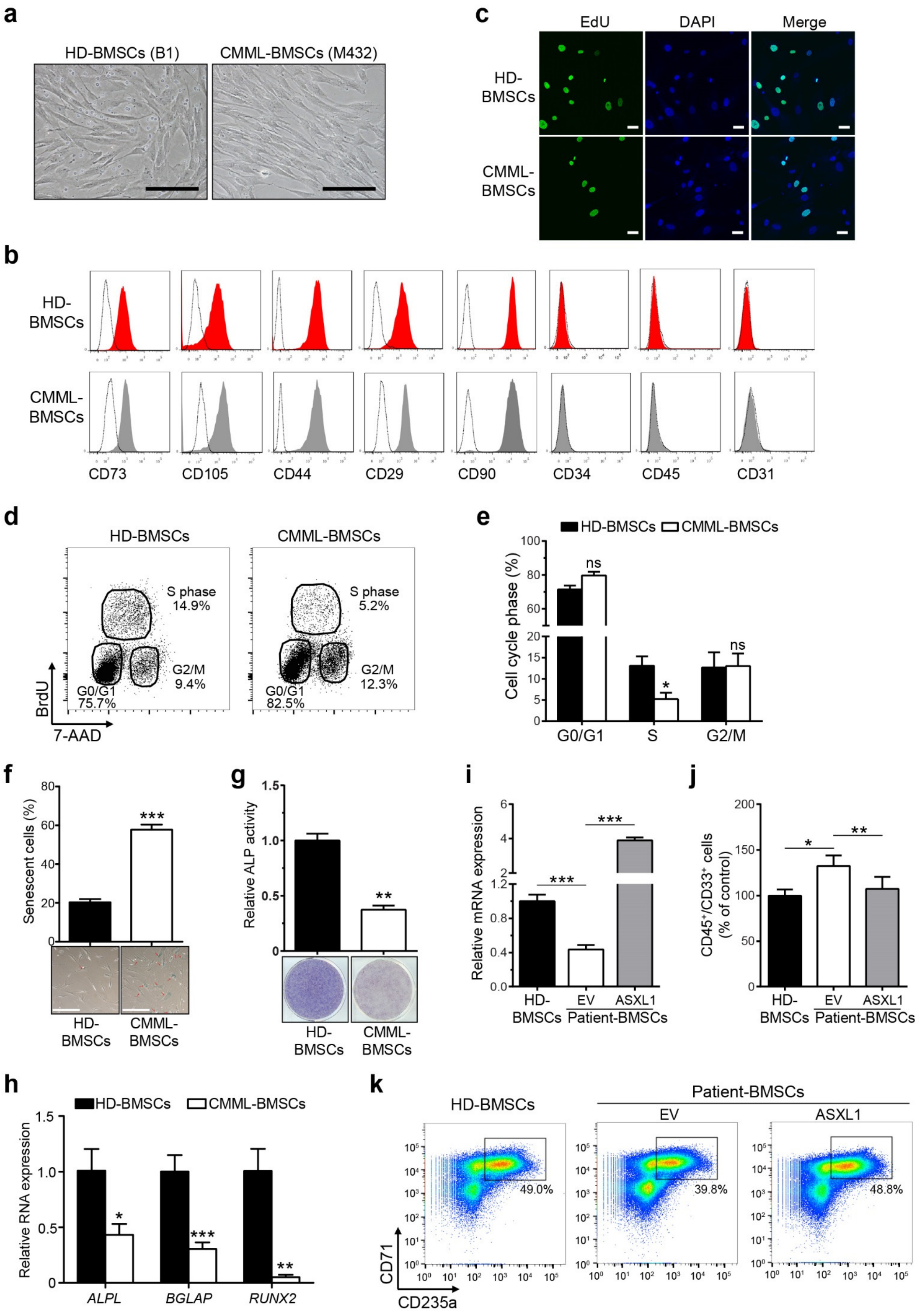

Fig. S1 Characterization of CMML-BMSCs and HD-BMSCs. Related to Fig. 1. (a) Representative images show the morphology of HD-BMSCs and CMML-BMSCs by light microscopy. Scale bar,

100 μm. (b) Representative figures of cell surface marker analysis in HD-BMSCs and CMML-BMSCs are shown. The colored histograms represent the BMSCs stained with different antibodies while the dot-line histograms represent the corresponding negative control stained with isotype-matched nonreactive fluorochrome-conjugated antibodies. (c) Representative micrographs of BMSCs after EdU incubation for 36 h are shown. Proliferating cells labelling by EdU (green), nuclear labelling by DAPI (blue) and the merged images are shown. Micrographs showed decreased proliferation potential of CMML-BMSCs versus HD-BMSCs. Scale bar, 40 μm. (d) BrdU assay indicates impaired cell proliferation ability of CMML-BMSCs. BMSCs were incubated with BrdU and 7-AAD, and analyzed by flow cytometry. Representative flow dot plots show the percentage of G0/G1, S phase and G2/M. (e) The bar graph shows the percentage of different cell cycle phases (n = 6 individual samples for HD, 4 for CMML). (f) The percentage of senescent cells is significantly increased in CMML-BMSCs compared with HD-BMSCs (n = 3 individual samples for HD, 5 for CMML). Representative images of HD-BMSCs and CMML-BMSCs after β-galactosidase staining are shown. The red arrows indicate the senescent cells. Blue staining represents the β-galactosidase-positive cells. Scale bar, 100 μm. (g) Alkaline phosphatase (ALP) staining shows significantly decreased osteogenic differentiation potential of CMML-BMSCs compared with HD-BMSCs (n = 4 individual samples for HD, 7 for CMML). (h) qPCR analysis shows decreased expression levels of the osteoblast marker genes in CMML-BMSCs compared with HD-BMSCs (n = 4 individual samples for HD, 7 for CMML). (i) qPCR showing the expression levels of *ASXL1* in MPN-BMSCs following overexpression of empty vector (EV) or *ASXL1* full-length cDNA (n = 2 individual samples for HD, 3 for MPN from two independent experiments). (j, k) The percentages of myeloid cells ($CD45^+/CD33^+$, j) and erythroid cells ($CD71^+/CD235a^+$, k) from co-culture of CB $CD34^+$ cells with HD-BMSCs and MPN-BMSCs overexpressing EV or *ASXL1* (n = 2 individual samples for HD, 3 for MPN from two independent experiments). Data represent mean ± s.e.m., ns, not significant, $^*P < 0.05$, $^{**}P < 0.01$, $^{***}P < 0.005$.

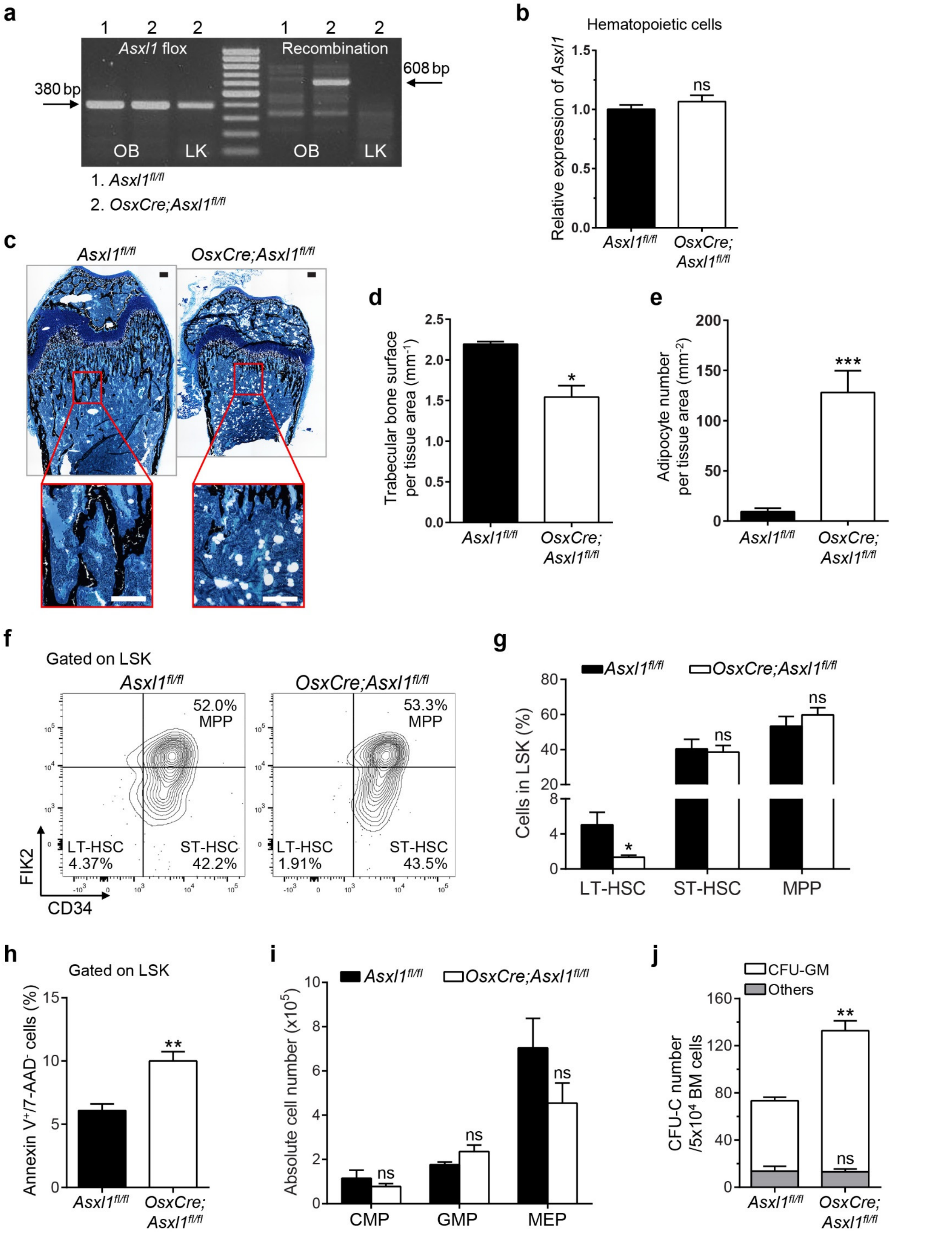

Fig. S2 Genotype and phenotype of *OsxCre;Asxl1$^{fl/fl}$* mice. Related to Fig. 2. (a, b) PCR genotyping (a) and qPCR (b) of Lin$^-$cKit$^+$ (LK) cells in *Asxl1$^{fl/fl}$* and *OsxCre;Asxl1$^{fl/fl}$* mice (n = 4

mice per genotype). OB, osteoblasts. (c) Representative photographs of the histology showing the decreased trabecular bone density and increased adipocyte number in *OsxCre;Asxl1*$^{fl/fl}$ mice. Scale bar, 100 μm. (d, e) Quantification of trabecular bone surface (d) and adipocyte numbers (e) in *Asxl1*$^{fl/fl}$ and *OsxCre;Asxl1*$^{fl/fl}$ mice (n = 5 mice per genotype). (f) Flow cytometric analysis of LT-HSC, ST-HSC, and MPP compartments in BM LSK cells of representative *Asxl1*$^{fl/fl}$ and *OsxCre;Asxl1*$^{fl/fl}$ mice. (g) Decreased frequency of LT-HSC in BM LSK cells of *OsxCre;Asxl1*$^{fl/fl}$ mice are shown (n = 4 mice per genotype). (h) Quantification of the apoptotic cells (Annexin V^{+}/7-AAD^{-}) within LSK cells of BM from *Asxl1*$^{fl/fl}$ and *OsxCre;Asxl1*$^{fl/fl}$ mice (n = 5 mice per genotype). (i) Absolute numbers of CMP, GMP and MEP cells are shown (n = 4 mice per genotype). (j) CFUs in BM cells from *Asxl1*$^{fl/fl}$ and *OsxCre;Asxl1*$^{fl/fl}$ mice were assessed in semi-solid media in the presence of mouse SCF, IL-3, EPO, TPO, GM-CSF and human IL-6 (n = 3~5 mice per genotype). Data represent mean ± s.e.m., ns, not significant, **P* < 0.05, ***P* < 0.01, ****P* < 0.005.

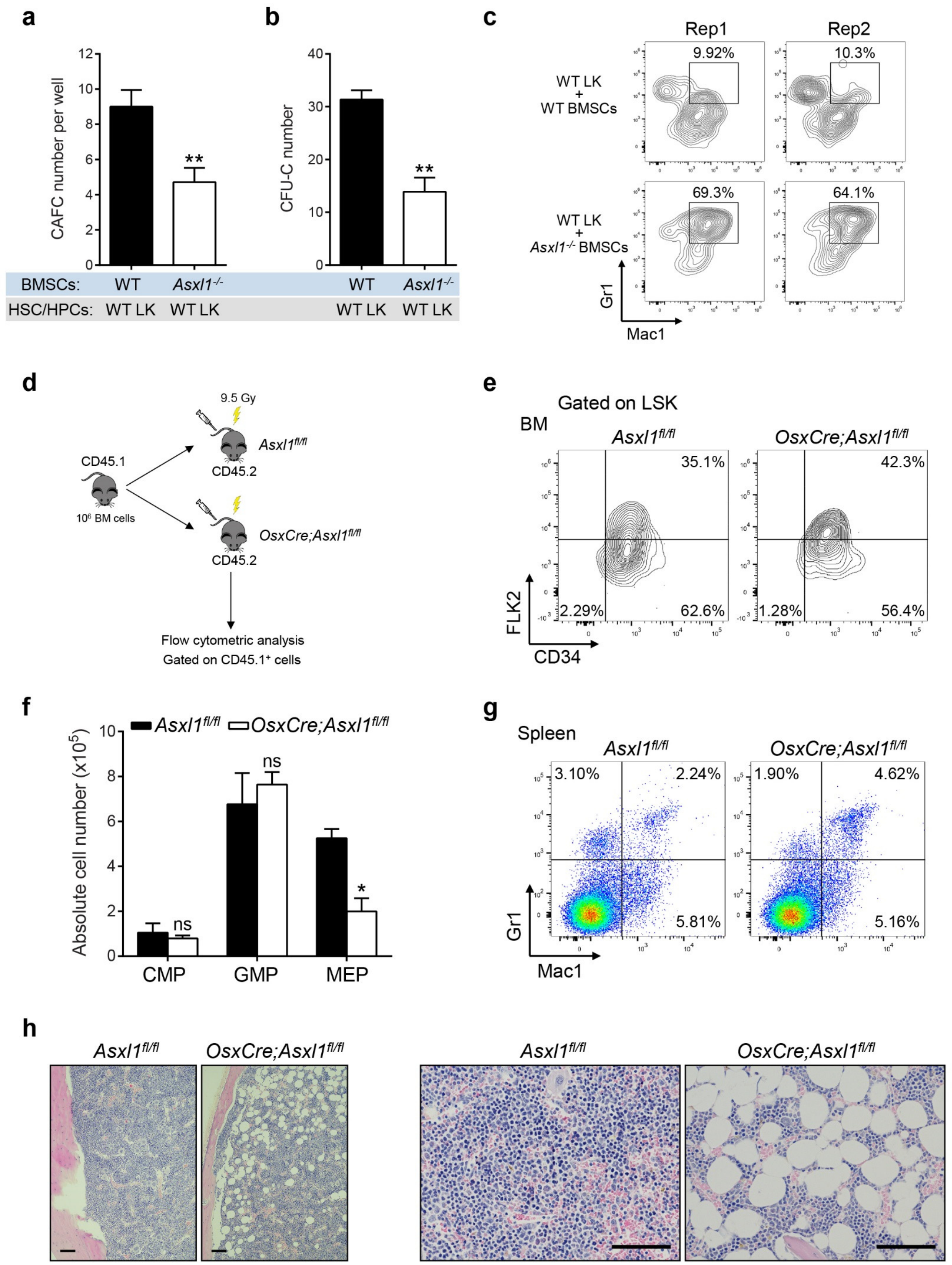

Fig. S3 Loss of *Asxl1* in the BM niche alters HSC/HPC fate. Related to Fig. 3. (a, b) *Asxl1*$^{-/-}$ BMSCs had reduced hematopoietic supportive activity compared with WT BMSCs (n = 3 mice per genotype). (c) After co-culture of WT LK cells with WT and *Asxl1*$^{-/-}$ BMSCs, the percentage of

Gr1$^+$/Mac1$^+$ cells was analyzed by flow cytometric analysis. (d) Schematic diagram of BM transplantation. CD45.1$^+$ BM cells from BoyJ mice (10^6 cells per recipient) were transplanted into lethally irradiated eight weeks old *Asxl1$^{fl/fl}$* and *OsxCre;Asxl1$^{fl/fl}$* recipients. After 5 months of transplantation, flow cytometric analysis of CD45.1$^+$ donor cells was performed in the recipient mice. (e) Flow cytometric analysis of LT-HSC, ST-HSC, and MPP compartments in BM CD45.1$^+$/LSK cells of representative *Asxl1$^{fl/fl}$* and *OsxCre;Asxl1$^{fl/fl}$* recipients. (f) Absolute numbers of CMP, GMP and MEP cells from *Asxl1$^{fl/fl}$* and *OsxCre;Asxl1$^{fl/fl}$* recipients are shown (n = 3 mice per genotype) (g) Flow cytometric analysis of Gr1$^+$/Mac1$^+$ cell populations in the spleen of representative *Asxl1$^{fl/fl}$* and *OsxCre;Asxl1$^{fl/fl}$* recipients. (h) Whole BM cells from *Asxl1$^{fl/fl}$* and *OsxCre;Asxl1$^{fl/fl}$* mice (10^6 cells per recipient) were transplanted into lethally irradiated eight weeks old WT recipients. Representative photomicrographs demonstrated BM histology of recipient mice 6 months post-transplantation at low (left) and high (right) magnification. Scale bar, 100 μm. Data represent mean ± s.e.m., ns, not significant, *$P < 0.05$, **$P < 0.01$.

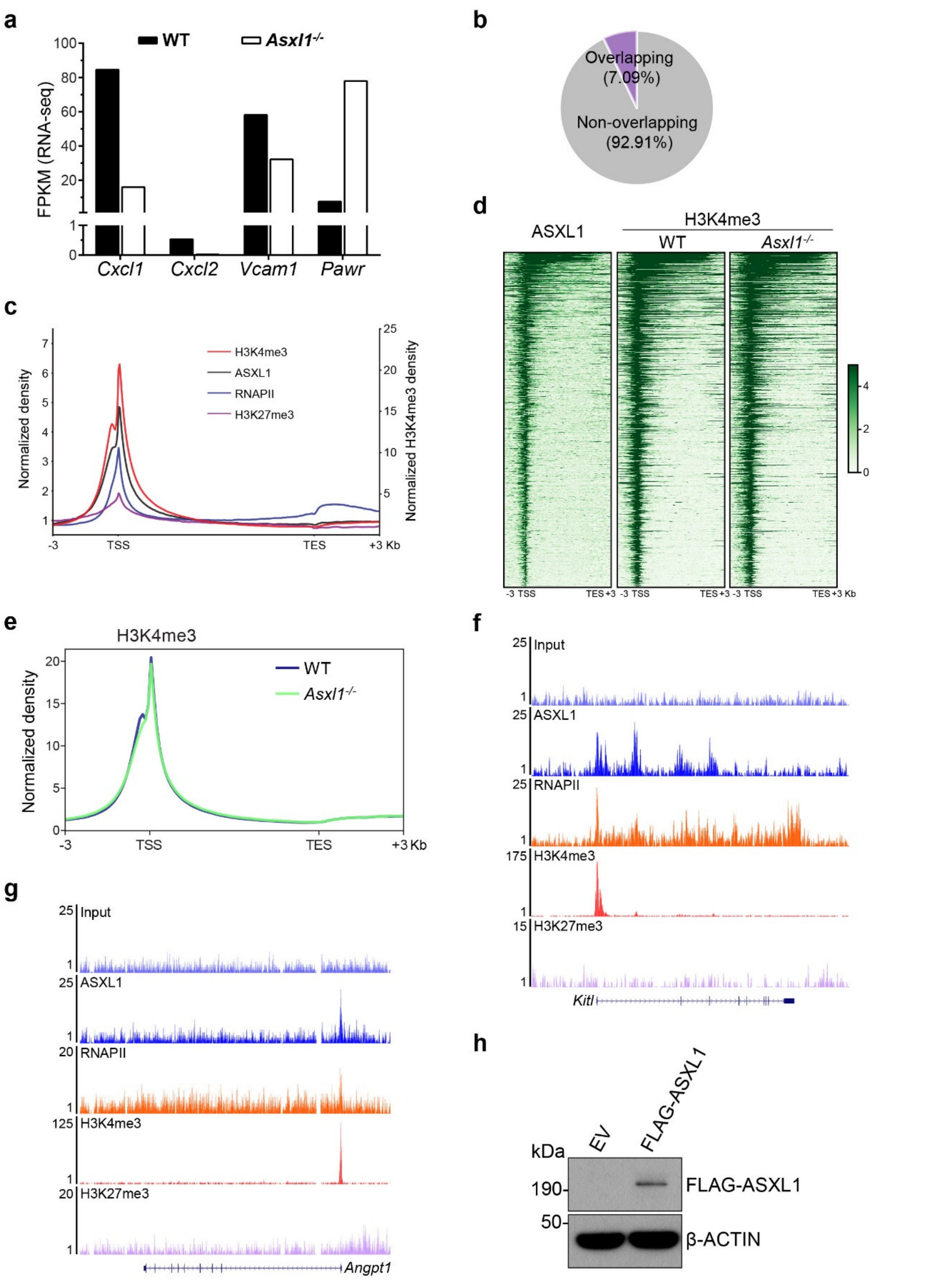

Fig. S4 RNA-seq and ChIP-seq analyses. Related to Fig. 4, 5 and 6. (a) Fragments per kilobase of transcript per million (FPKM) values of differentially expressed genes in RNA-seq are shown.

Bars represent the mean of two replicates. (b) Pie graph shows the percentage of overlapping peaks of ASXL1 with H3K27me3 in WT BMSCs. (c) Average genome-wide occupancies of H3K4me3 (red, right y axis), ASXL1 (black), RNAPII (blue) and H3K27me3 (purple) in WT BMSCs on all genes along the transcription unit. (d) Heatmaps of ASXL1 and H3K4me3 on ASXL1-binding genes in WT and *Asxl1*$^{-/-}$ BMSCs. The genes ranked from highest to lowest ASXL1 level. (e) Average genome-wide occupancies of H3K4me3 in WT and *Asxl1*$^{-/-}$ BMSCs. (f, g) Representative genome browser tracks showing ASXL1, RNAPII, H3K4me3 and H3K27me3 occupancies on regions of *Kitl* and *Angpt1*. (h) Western blotting showing the ASXL1 expression using anti-FLAG antibody in WT BMSCs overexpressed FLAG-ASXL1.

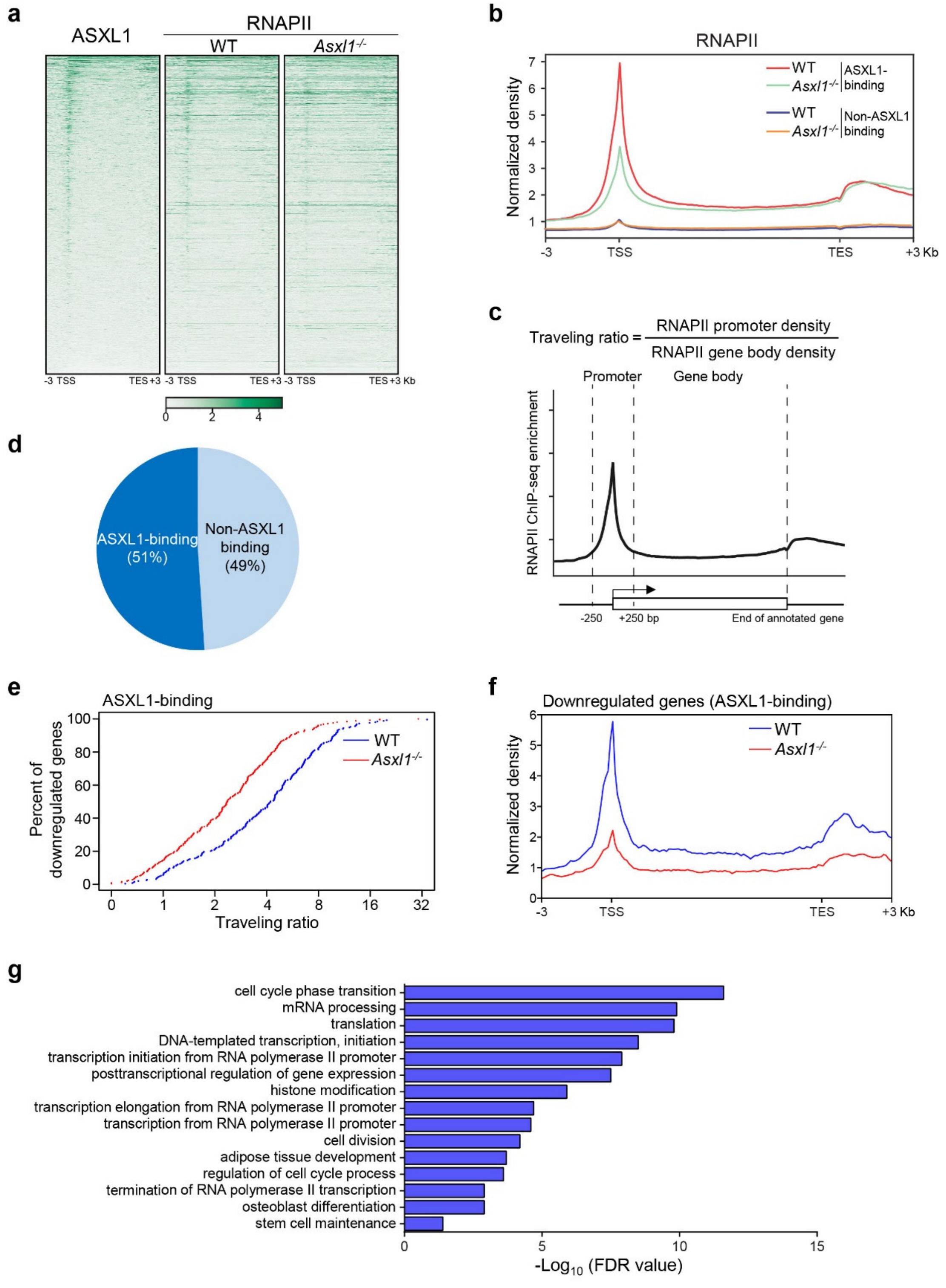

Fig. S5 Loss of *Asxl1* dysregulates gene expression through RNAPII transcriptional activity. Related to Fig. 7. (a) Heatmaps of ASXL1 and RNAPII on non-ASXL1 binding genes in WT and *Asxl1*$^{-/-}$ BMSCs. (b) Loss of *Asxl1* in BMSCs altered RNAPII occupancy mainly on ASXL1-binding

genes. (c) Schematic representation describing the calculation used to determine the traveling ratio (TR) at each RNAPII-bound gene in BMSCs. (d) Pie graph showing 51% of downregulated genes are ASXL1-binding genes. (e) RNAPII TR calculations of ASXL1-bound downregulated genes in WT and *Asxl1*$^{-/-}$ BMSCs. (f) Average genome-wide occupancies of RNAPII in WT and *Asxl1*$^{-/-}$ BMSCs on ASXL1-bound downregulated genes along the transcription unit. (g) The Gene ontology analysis for reduced RNAPII enrichment of ASXL1-binding genes is shown.

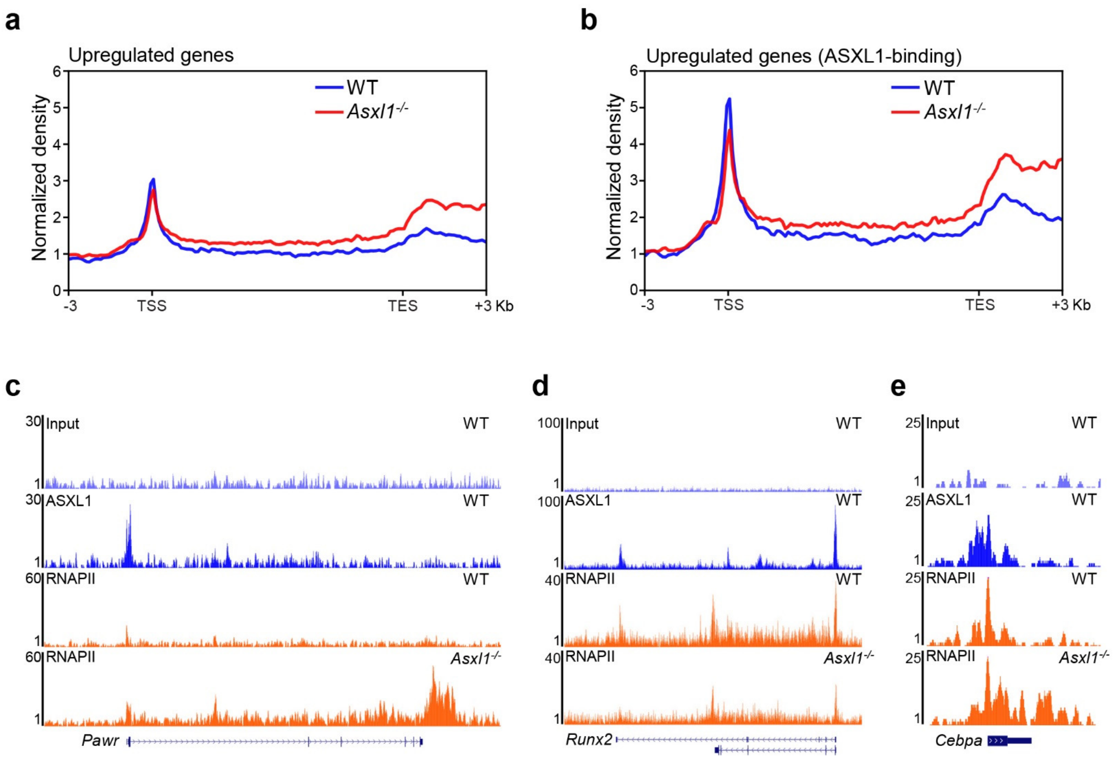

Fig. S6 Loss of *Asxl1* upregulates gene expression through RNAPII transcriptional activity. Related to Fig. 7. (a, b) Average genome-wide occupancies of RNAPII in WT (blue) and *Asxl1*$^{-/-}$ (red) BMSCs on all (a) or ASXL1-binding (b) upregulated genes along the transcription unit. (c-e) Representative genome browser tracks showing ASXL1 and RNAPII occupancy on regions of *Pawr*, *Runx2*, and *Cebpa*.

Table S1. Clinical information of CMML patients.

Patients	Gender	Diagnosis	Age at diagnosis	CPSS-P*
M6	M	CMML	66	Intermediate-2
M73	F	CMML	57	High
M97	M	CMML	62	Intermediate-1
M144	M	CMML	72	Intermediate-1
M260	M	CMML	78	High
M277	M	CMML	87	Intermediate-1
M288	M	CMML	81	Intermediate-2
M340	F	CMML	63	Low
M347	M	CMML	29	Intermediate-1
M368	M	CMML	61	Intermediate-2
M429	F	CMML	57	Intermediate-2
M432	M	CMML	42	Intermediate-1
M479	M	CMML	75	Intermediate-2

* Modified CMML-specific prognostic scoring system including platelet count (CPSS-P).

Table S2. Flow antibodies used in this study.

Antibody name	Clone	Color	Catalog	Company
Mouse anti-human CD45	clone HI30	FITC	555482	BD PharMingen
Mouse anti-human CD34	clone 581	APC	555824	BD PharMingen
Mouse anti-human CD33	clone P67.6	APC	340474	BD PharMingen
Anti-human CD235a	clone HIR2	PE	12-9987-80	eBioscience
Anti-human CD71	CY1G4	APC-Cy7	334109	eBioscience
Mouse anti-human CD73	AD2	PE	550257	BD PharMingen
Mouse anti-human CD105	266	APC	562408	BD PharMingen
Mouse anti-human CD44	C26	APC-H7	560532	BD PharMingen
Mouse anti-human CD29	MAR4	PE-Cy5	559882	BD PharMingen
Rat anti-Mouse CD34	RAM34	FITC	553733	BD PharMingen
Anti-mouse CD34	RAM34	Alexa Fluor 700	56-0341-82	eBioscience
Rat anti-Mouse Ly-6A/E (Sca1)	D7	PE-Cy7	558162	BD PharMingen
Rat anti-Mouse CD117	2B8	PE	553355	BD PharMingen
Rat anti-Mouse CD117	2B8	APC	553356	BD PharMingen
Rat anti-Mouse CD117	2B8	PerCP-Cy5.5	560557	BD PharMingen
Anti-Mouse CD117	2B8	FITC	17-1171-82	eBioscience
Rat anti-Mouse CD16/CD32	2.4G2	APC-Cy7	560541	BD PharMingen
Rat anti-mouse CD135	A2F10.1	BV421	562898	BD Horizon
Mouse Lineage Antibody Cocktail		APC	51-9003632	BD PharMingen
Rat anti-Mouse CD71	C2	FITC	553266	BD PharMingen
Rat anti-Mouse TER-119	TER-119	APC	557909	BD PharMingen
Rat anti-Mouse Ly-6G and Ly-6C	RB6-8c5	PerCP-Cy5.5	552093	BD PharMingen
Rat anti-Mouse Ly-6G and Ly-6C	RB6-8c5	PE-Cy7	552985	BD PharMingen
Rat anti-Mouse CD11b	M1/70	PE	553311	BD PharMingen
Mouse anti-Mouse CD45.2	104	PerCP-Cy5.5	552950	BD PharMingen
Mouse anti-Mouse CD45.1	A20	FITC	553775	BD PharMingen
Annexin V		PE	51-65875X	BD PharMingen
7-AAD		PerCP-Cy5.5	51-68981E	BD PharMingen

Table S3. Primers used for in this study.

Gene Name	Forward	Reverse
Human		
ASXL1	CCACAGCCCACTAAAGAGGA	CAGAGCACGGGCTTTAATGT
ALPL	GATGTGGAGTATGAGAGTGACG	GGTCAAGGGTCAGGAGTTC
RUNX2	GTTAATCTCCGCAGGTCACTAC	GATGAGGAATGCGCCCTAAA
BGLAP	CAGCGAGGTAGTGAAGAGAC	TGAAAGCCGATGTGGTCAG
GADPH	GAAGGTGAAGGTCGGAGTC	GAAGATGGTGATGGGATTTC
Mouse		
Asxl1	TCACACCGAAAAGCCACAG	GGGCATATCTGGTAAGTGGG
Cxcl1	AACCGAAGTCATAGCCACAC	CAGACGGTGCCATCAGAG
Cxcl2	AATGCCTGAAGACCCTGC	TTTTGACCGCCCTTGAGAG
Vcam1	GACCTGTTCCAGCGAGGGTCTA	CTTCCATCCTCATAGCAATTAAGGTG
Pawr	CAGGACAGAAGGAACGGAAG	GGTTGTGCTTTTGTATCTGCC
Actb	GGCTGTATTCCCCTCCATCG	CCAGTTGGTAACAATGCCATGT
